# Uncommon ileal perforation due to intestinal tuberculosis: A case report and literature review

**DOI:** 10.1097/MD.0000000000041099

**Published:** 2025-01-03

**Authors:** Jianhua Ju, Jingyu Liu, Wei Dong, Yuxu Zhong, Haibo Chu

**Affiliations:** a Department of General Surgery, Jiaozhou Branch of Shanghai East Hospital, Tongji University, Qingdao, China; b State Key Laboratory of Toxicology and Medical Countermeasures, Beijing Institute of Toxicology and Pharmacology, Beijing, China.

**Keywords:** anti-tubercular therapy, diagnostics, ileal perforation, intestinal tuberculosis, surgical operation

## Abstract

**Rationale:**

Tuberculosis (TB) is a chronic granulomatous infectious disorder, caused by *Mycobacterium tuberculosis*. Extrapulmonary TB, which accounts for 20% of cases, includes intestinal TB in 10%. Gastrointestinal TB leads to intestinal perforation in 4% to 7.6% of cases, with a mortality rate of 30%.

**Patient concerns:**

We conducted a retrospective analysis of a patient with ileal perforation due to intestinal TB. A male in his early 20s (initial weight, 35 kg) presented with a 2-day history of abdominal pain, exhibiting tenderness, rebound tenderness, and muscular guarding upon physical examination. Computed tomography (CT) imaging revealed a significant amount of free gas and fluid in the abdominal cavity. Subsequently, the patient underwent ileal repair and ileostomy.

**Diagnoses:**

Histopathological examination confirmed multifocal amorphous pink caseating necrotic material and Langhans giant cells in the mesenteric lymph nodes. A polymerase chain reaction (PCR) assay confirmed infection with *M tuberculosis*.

**Interventions:**

On the 20th postoperative day, enteral nutrition was initiated concomitantly with antitubercular therapy (ATT). After 1 month, enteral nutrition and oral diet were alternated for 2 months, then changed to oral diet alone, and the patient was discharged to continue ATT. Five months later, the patient’s weight increased by 20 kg, and he began exercising outdoors. The patient underwent a successful ostomy reversal.

**Outcomes:**

At the 12-month follow-up, his body weight had increased to 65 kg, PCR testing was negative for *M tuberculosis*, and antituberculosis drugs were discontinued.

**Lessons:**

This case highlights the successful management of ileal perforation due to intestinal TB with peritonitis, without complications such as fistulas. Diagnosis of small bowel perforations due to intestinal TB remains challenging even for experienced clinicians, and surgical approaches are controversial. We emphasized the significance of diagnostics of such cases and often requiring a multidisciplinary approach involving various medical teams.

## 1. Introduction

Tuberculosis (TB) is a chronic granulomatous infectious disorder caused by *Mycobacterium tuberculosis*, with the primary route of infection involving inhalation of Mycobacterium-impregnated airborne droplets.^[1]^ TB is a leading infectious cause of global mortality, second only to coronavirus disease 2019 (COVID-19). It resulted in approximately 10.6 million new patients and1.6 million deaths in 2021 globally, up from 1.5 million in 2020 to 1.4 million in 2019.^[2]^ These statistics suggest that the COVID-19 pandemic disrupted decades of global progress in decreasing TB mortality, and the total number of TB-related deaths in 2020 has reverted to the same level observed in 2017.^[2]^ According to the 2020 World Health Organization report, 9.9 million people worldwide are infected with TB, with the majority residing in Southeast Asia. While TB typically affects the pulmonary system, comprising 50% of cases, the incidence of abdominal TB is increasing in developed countries, and abdominal TB currently accounts for approximately 10% of cases. The abdomen is the most common extrapulmonary site for TB, where the organism can infect the gastrointestinal tract, peritoneum, viscera, lymph nodes and other structures.^[3]^

Intestinal perforation resulting from gastrointestinal TB occurs in 4% to 7.6% of cases, carrying a mortality rate of 30%.^[4]^ Due to this high mortality rate, it is important to recognize the clinical manifestations of abdominal TB, along with the major risk factors, which include a history of TB infection and residence in or travel to TB-endemic regions.^[5]^ Typically, ulcers and strictures affect the small intestine, while ulcerohyperplasia is common in the colon and ileocecal region.^[6]^ The ileocecal junction or the jejunoileum are involved in approximately 36% to 90% of abdominal TB cases, primarily due to the high density of lymphoid aggregates and physiologic stasis in these segments, and peritoneal involvement follows in 33% of cases.^[7]^ Free perforation, occurring in 1% to 10% of patients, is 1 of the most feared complications of intestinal TB, with the terminal ileum being the most common site, and the majority (90%) of perforations being solitary.^[8]^

Diagnosis of abdominal TB remains challenging due to its diverse clinical presentation, often mimicking other conditions, meaning that diagnosis can be delayed by weeks or months. Treatment also presents multiple obstacles. Surgery can be difficult due to development of a frozen in the abdominal cavity, and between 6% and 30% of patients receiving anti-tubercular therapy (ATT) experience a paradoxical reaction, which may be a delayed hypersensitivity response to bacterial antigens. In addition, intestinal perforation occurs in an estimated 1% to 15% of all patients with intestinal TB, highlighting the complexities in managing this disease.^[4]^ Here, we present a case of ileal perforation due to intestinal TB from our institution, accompanied by a review of relevant literature.

## 2. Case presentation

**Figure 1.**
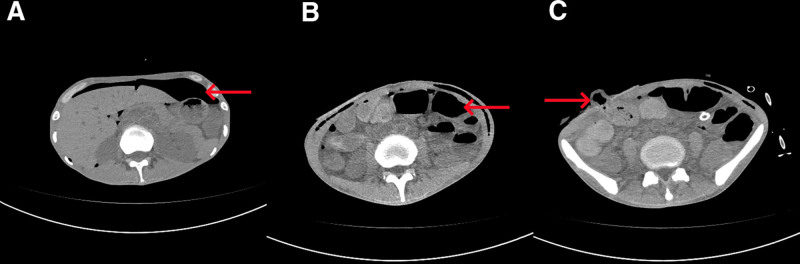
Computed tomography (CT) imaging of ileal perforation. The first pre-operation examination in the axial position revealed a large amount of free gas (A) and the second pre-operation and post-operation examination in the axial positions (B and C) revealed small bowel dilation, gas–liquid level, seroperitoneum, and the stoma location in the abdominal wall (indicated by arrows).

An male patient in his early 20s presented to our hospital on April 4, 2023, with a 2-day history of abdominal pain. The patient had no prior history of pulmonary TB. The patient weighed 35 kg and was 1.7 m tall, and physical examination revealed that the patient was malnourished, with pallor and non-palpable superficial lymph nodes. The patient presented with tenderness, rebound tenderness, and muscular guarding throughout the abdomen. Bowel sounds were absent. Laboratory findings showed a white blood cell count of 11.2 × 10^9^/L (normal range: 3.5–9.5 × 10^9^/L), neutrophil count of 97.9% (normal range: 50% to 70%), and hemoglobin, albumin,interleukin-6, and procalciton in levels of 42g/L, 17.5 g/L, 66.30 pg/mL, and 0.606 ng/mL, respectively (normal ranges: 120–160 g/L; 35–50 g/L; 37.3 to 46.3 pg/mL; and <0.05 ng/mL, respectively). Computed tomography (CT) imaging of the abdomen revealed a large amount of free gas and fluid in the abdominal cavity (Fig. 1A). The patient was diagnosed with perforation of the digestive tract.

**Figure 2.**
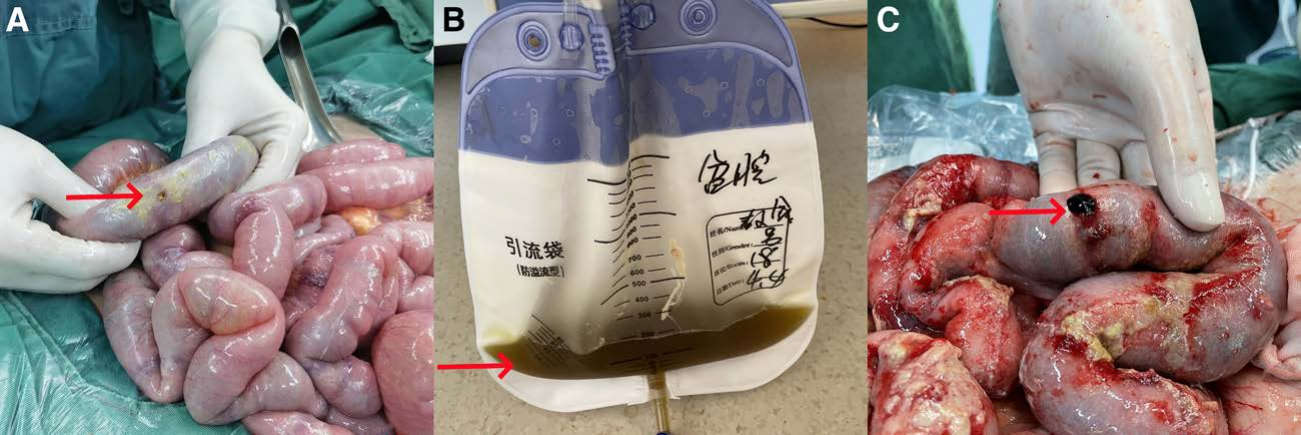
Intraoperative and postoperative conditions. (A) The first intraoperative findings revealed a 0.5 cm diameter crevasse in the ileum 30 cm from the ileocecal valve. (B) In the second pre-operation, light green fluid was extracted from the pelvic drainage tube. (C) The second intraoperative findings revealed a 0.3 cm diameter crevasse 70 cm away from the ileostomy (indicated by arrows).

On April 4, a laparoscopic exploration was performed, revealing yellow purulent fluid (1000 mL) in the abdominal cavity, intestinal edema, and segmental thickening with multiple patchy ulcers invading the muscle layer in the ileum 80 cm from the ileocecal valve, with the small intestine measuring 3.4 m in length. A 0.5 cm diameter crevasse was found in the ileum 30 cm from the ileocecal valve, with overflowing intestinal fluid and regional mesenteric lymph node enlargement (Fig. 2A). Ileal repair and ileostomy were performed, and a pelvic drainage tube was inserted. On the 4th postoperative day, the patient developed a fever with a temperature of 39.5 °C, and light green fluid was extracted from the pelvic drainage tube at a rate of 100 mL/24h (Fig. 2B). Repeat CT imaging of the abdomen showed a large amount of free gas and fluid (Fig. 1B and C).

**Figure 3.**
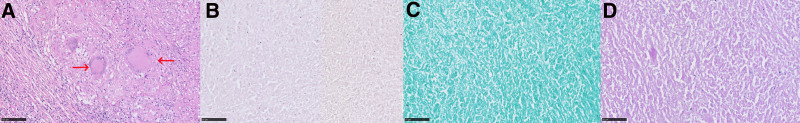
HE staining and special staining stained histological slides. (A) Multifocal caseating necrosis accompanied by Langhans giant cells in the lymph nodes (indicated by arrows, HE × 200, Scale bars = 50 μm). (B) Negativity of acid-fast bacilli staining (AFB × 200, Scale bars = 50 μm); (C) Negativity of gomori methenamine silver staining (GMS × 200, Scale bars = 50 μm); (D) Negativity of PAS-naphtholsulfone S staining (PAS × 200, Scale bars = 50 μm). AFB = acid-fast bacilli, GMS = gomori methenamine silver, HE = hematoxylin-eosin, PAS = periodic acid–schiff–naphtholsulfone S.

Emergency open surgery was performed on the 5th postoperative day, revealing a 0.3 cm diameter crevasse 70 cm away from the ostomies, inflammatory changes in the intestinal canal, increased abdominal pressure, and darkened intestinal canal color (Fig. 2C). The crevasse was repaired with 3 to 0 absorbable wire, 2 Lee double cannulas were placed in the abdominal cavity, and the incisions were covered with a polypropylene mesh. Histopathological examination confirmed multifocal amorphous pink caseating necrotic material and Langhans giant cells in the lymph nodes (Fig. 3A). Acid-fast bacilli (AFB) staining, Gomori methenamine silver (GMS) staining, and periodic acid–schiff–naphtholsulfone S (PAS) staining were negative (Fig. 3B–D), but *M tuberculosis* was detected in the mesenteric lymph node by polymerase chain reaction (PCR). On the 7th postoperative day, 1000 mL of bright red fluid accompanied by a blood clot was discharged from the stoma due to bleeding from a stress ulcer in the small intestine.

**Figure 4.**
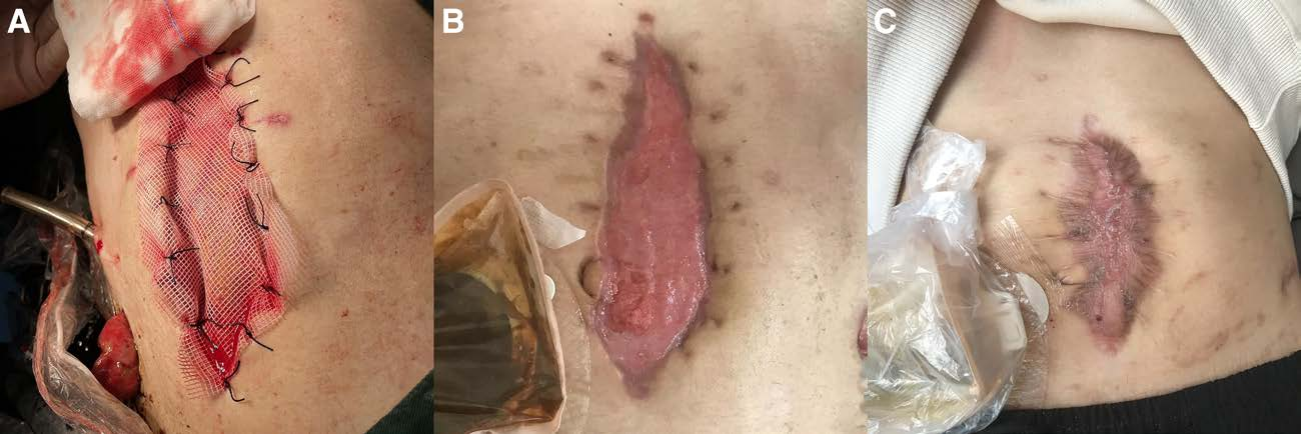
Incision opening and healing conditions. (A) The abdomen was left open, and the intestinal tube was covered with a polypropylene mesh. (B) The surface of the intestinal tube showed fresh granulation tissues. (C) The incision was healed with skin scar tissues.

On the 20th day, enteral nutrition was initiated concomitantly with an ATT regimen that consisted of an intensive phase with 1 month of isoniazid 100 mg qd, rifampicin 125 mg qd, and ethambutol 85 mg tid. After 1 month, enteral nutrition and oral diet were alternated for 2 months, then changed to oral diet alone, and the patient was discharged to continue a maintenance phase of ATT, which consisted of isoniazid 100 mg qd and rifampicin 125 mg qd (Fig. 4–C). Five months later, the patient’s weight had increased by 20 kg, and he began exercising outdoors. Laboratory findings showed a white blood cell count of 9.52 × 10^9^/L (normal range: 3.5–9.5 × 10^9^/L), neutrophil count of 74% (normal range: 50% to 70%), a hemoglobin level of 130 g/L (normal range: 120–160 g/L), and an albumin level of 43 g/L (normal range: 35–50 g/L), with normal liver and kidney functions. The patient underwent a successful ostomy reversal. At the 12-month follow-up, his body weight had increased to 65 kg, PCR testing for *M tuberculosis* was negative, and anti-TB drugs were discontinued. The test results of the patient in prior- and post-treatment are summarized in Table 1.

**Table 1:** Laboratory results of the patient with tubercular intestinal perforation.

Variables	Patient’s values	Reference values
Prior-treatment		
White blood cell count	11.2 × 10^9^/L	3.5 to 9.5 × 109/L
Predominantly neutrophils	97.9%	50% to 70%
Hemoglobin	42 g/L	120 to 160 g/L
Albumin	17.5 g/L	35 to 50 g/L
Interleukin-6	66.30 pg/mL	37.3 to 46.3 pg/mL
Procalcitonin	0.606 ng/mL	<0.05 ng/mL
C-reactive protein	235 mg/dL	0 to 19 mg/dL
Tuberculosis-PCR testing	Positive	Negative
Posttreatment		
White blood cell count	9.52 × 10^9^/L	3.5 to 9.5 × 109/L
Predominantly neutrophils	74%	50% to 70%
Hemoglobin	130 g/L	120 to 160 g/L
Albumin	43 g/L	35 to 50 g/L
Interleukin-6	28.24 pg/mL	37.3 to 46.3 pg/mL
Procalcitonin	0.01 ng/mL	<0.05 ng/mL
C-reactive protein	15 mg/dL	0 to 19 mg/dL
Tuberculosis-PCR testing	Negative	Negative

All patient-identifying information has been removed. The study was approved by Jiaozhou Branch of Shanghai East Hospital, Tongji University Ethics Committee. The patient provided written consent for both treatment and publication of this report. The reporting of this study conforms to CARE guidelines.^[9]^

## 3. Discussion

In Western countries, nontraumatic ileal perforation commonly occurs due to malignancies, Crohn disease, hernias, radiotherapy, adhesive bands, and other mechanical causes. In contrast, in developing countries, ileal perforation is often caused by infectious diseases such as typhoid fever and TB. The unique anatomical structure and high absorption rate of the ileocecal region make it a common site for intestinal perforation. Ileal perforation is a serious complication of abdominal TB, with studies reporting mortality rates of up to 30%.^[10]^ Even in developing countries, intestinal perforation is relatively uncommon and usually occurs due to the reactivation of a dormant focus. Tuberculous perforations are mainly solitary and located immediately proximal to the site of stricture although multiple perforations are reported but uncommon.^[11]^ The clinical features of tuberculous perforation are nonspecific, with common symptoms including intermittent abdominal pain, vomiting, constipation, and uncommon symptoms that include melenic stool. Sudden abdominal pain with distension suggests intestinal perforation. Most patients exhibit emaciation, and severe cases may present with toxemia, including tachycardia and hypotension. Abdominal examination yields findings that are typical of peritonitis, including diffuse tenderness and stiffness.

Intestinal TB, caused by *M tuberculosis*, is a chronic, specific infection that accounts for 1% to 3% of all TB cases.^[1]^ Primary abdominal TB involves the direct invasion of abdominal organs and tissues by *M tuberculosis* and is typically seen in children and immunocompromised individuals. Secondary abdominal TB results from the spread of TB through the bloodstream or lymphatic system to the abdominal organs and is more common in adults and immunocompromised individuals.^[12]^ Complications of intestinal TB include intestinal obstruction, perforation, intestinal fistula, abdominal effusion, and gastrointestinal bleeding. Its clinical manifestations are atypical of TB, making it difficult to distinguish from other diseases and prone to misdiagnosis. Weight loss and mild-to-moderate anemia in patients with intestinal TB are associated with chronic inflammatory abscesses, reduced intake, and impaired absorption.^[13]^ Intestinal bleeding caused by intestinal TB is rare and may be attributed to ulcers developing from endarteritis. Bleeding episodes in patients with diffuse ulceration in a long bowel segment can have a poor prognosis within a month following surgery.^[14]^

Previous research has demonstrated that in patients receiving ATT, a paradoxical reaction to the anti-TB treatment and poor nutritional status are factors that contribute to intestinal perforation.^[15]^ Ramesh et al^[16]^ found the incidence of intestinal TB in the ileocecal area to be approximately 17% to 42%. Cheng et al^[17]^ retrospectively studied 85 patients with intestinal TB and found that 75 cases (88.2%) had chronic pain around the umbilical site and right lower abdomen, and of these 75 cases, 21 were complicated by intestinal perforation. Morgan et al^[18]^ reported a patient with small bowel perforation near the ileocecal valve and sigmoid perforation due to miliary TB, and multiple granulomatous lesions over the small intestine that were nearing perforation. Other risk factors for intestinal TB include cirrhosis, HIV infection, diabetes, potential malignancy, malnutrition, and treatment with antitumor necrosis factor agents, corticosteroids, and continuous peritoneal dialysis.

The differential diagnosis for TB peritonitis should include carcinomatosis, ovarian cancer, mesothelioma, or non-TB peritonitis. Distinguishing between Crohn disease and intestinal TB can be challenging, as their clinical, radiological, endoscopic, and histological features overlap. Crohn disease patients with spontaneous intestinal perforation are typically younger than those with intestinal TB, however. Nocturnal hyperhydrosis is common in intestinal TB, while diarrhea is more common in Crohn disease.^[19]^ End-stage liver disease can be complicated by ascites and spontaneous bacterial peritonitis and thus can also lead to symptoms similar to TB peritonitis. Our case further demonstrated the importance of using a combination of clinical findings, laboratory examinations, etiological investigations, and imaging studies for accurate diagnosis. The patient in this case, a male patient in his early 20s with no history of pulmonary TB, presented with emaciation, anemia, severe malnutrition, and mental fatigue. Physical examination revealed a scaphoid abdomen, tenderness, rebound pain, and absent bowel sounds. Postoperative pathology confirmed intestinal TB. A chest X-ray showed no abnormalities, and AFB staining and sputum culture results were negative, indicating primary intestinal TB.

Ultrasound is a noninvasive modality that can differentiate between solid and cystic lesions and detect free and loculated ascites, thickening of the omentum and peritoneum, lymphadenopathy, and gut thickening. In patients with suspected abdominal TB, ultrasound can reveal ascites with echogenic debris, peritoneal and omental thickening, intestinal wall thickening, and lymphadenopathy. However, the reliability of ultrasound as a diagnostic tool for specific features of abdominal TB is limited due to its high false-negative rate and the subjectivity of human manipulation and interpretation, leading to the potential to miss subtle signs.^[20]^ Abdominal CT can confirm ultrasound findings and assess disease dissemination. It can also help differentiate peritoneal TB from peritoneal cancer, which is characterized by peritoneal thickening, lymph node enlargement with central low density, and calcification. CT can be used to assess inflammation, adhesions, including intestinal obstructions, perforations, abscesses, and fistulas, which are important to investigate prior to surgical intervention. Enhanced CT may indicate intestinal wall thickening, asymmetric thickening of the ileocecal valve, strictures in the distal ileum, and necrotic enlarged lymph nodes in the mesentery.^[21]^ In fact, the ability of enhanced CT to detect thickening of the affected portion of the intestinal tract has been found to exhibit a sensitivity of 87% and a specificity of 47.4%, and its sensitivity of detection of abdominal lymphadenopathy was 66%, with as pecificity of 36.8%.^[22]^ CT findings are often nonspecific and include peritoneal thickening, ascites, lymphadenopathy, hepatomegaly, hepatic or splenic lesions, and calcifications. The diagnostic rate of CT for visceral or peritoneal TB ranges from 69% to 78%, highlighting the challenges in diagnosing extrapulmonary TB. In short, while CT is a valuable tool for diagnosing abdominal TB, making a definitive diagnosis can be difficult.^[23]^

Due to the similarities in clinical manifestations and abdominal signs between Crohn disease and intestinal TB, misdiagnosis and mistreatment can occur. Axial CT enterographic image findings in TB, such as segmental small bowel involvement, mural stratification, comb sign, and fibrofatty proliferation are more commonly associated with Crohn disease, whereas mesenteric lymph node changes, including calcification or central necrosis, and focal ileocecal lesions are frequently seen in intestinal TB.^[24]^ In our case, the patient presented with nonspecific abdominal pain symptoms, and the diagnosis of gastrointestinal perforation was considered based on CT findings upon admission. However, abdominal CT only revealed peritoneal free gas and fluid, with typical ileocecal wall thickening and segmental stenosis not evident. Combining endoscopy with CT can be beneficial in examining the gastrointestinal tract and evaluating the extent of disease and therapeutic response in patients with intestinal TB or Crohn disease.

The characteristics of intestinal TB include chronic granulomatous inflammation in the gastrointestinal tract, characterized by a collection of vaguely contoured epithelioid histiocytes (macrophages) that are typically large (>200 μm), confluent, dense (>5–10/hpf), and located in the submucosa, often with central caseating changes. Other features include submucosal granulomas, ulcers lined with epithelioid tissue cells, and disproportionate submucosal inflammation. In the early stages of intestinal TB, there are no pathognomonic features, and endoscopy may reveal intestinal inflammation, with biopsies having a poor yield even in ulcerated lesions, rarely showing granulomas and often displaying nonspecific inflammatory lesions. The diagnosis can be confirmed if histological examination of peri-intestinal lymph nodes demonstrates caseating necrosis.^[25]^

The pathological types of intestinal TB include hyperplastic, ulcerative, mixed, or strictured types. Hyperplasia may lead to a mass or stricture of the intestinal cavity, resulting in intestinal obstruction. Intestinal perforation may occur in the ulcerative type, particularly in immunocompromised patients. Ninety percent of intestinal perforations are isolated.^[10]^ Histological characteristics of intestinal TB include granulomas with caseating necrosis, conglomerate epithelioid histiocytes, and disproportionate submucosal inflammation. Caseating necrosis has been observed in 13% to 33% of cases, and the presence of granulomas facilitates the detection of 57% to 74% of intestinal TB cases.^[26]^

The high incidence of caseating granuloma among intestinal TB cases facilitates its diagnosis. Cheng et al^[17]^ reported that among 49 cases with endoscopic biopsies and 36 cases with surgical specimens, 69.4% and 72.2% respectively exhibited caseating granulomas. The positive rate of caseating necrosis was lower than that of caseating granuloma, with endoscopic biopsies at 20.4% and surgical specimens at 30.6%. Gan et al^[27]^ analyzed 81 cases of intestinal TB from January 2010 to December 2012, of which 6 cases (7.4%) were complicated by intestinal perforation. The pathological results showed chronic mucositis in 48 cases (87.3%), ulceration in 41 cases (74.5%), caseating granuloma in 41 cases (74.5%), lymphocyte aggregation in 38 cases (69.1%), granulomatous fusion in 32 cases (58.2%), submucosal narrowing or shrinkage in 15 cases (27.3%), caseating necrosis in 14 cases (25.5%), and crypt inflammation or crypt abscess in 3 cases (5.5%). Ma et al^[24]^ noted significant differences in the size, number, location, and patterns of granulomas found in intestinal TB and in Crohn disease, and they also detailed histopathologic features that characterize these 2 disorders.

Several molecular assays have also been utilized successfully to diagnose intestinal TB. For example, immune staining of cell surface markers has proven useful. Interferon-γ release assay and TB-PCR analysis have achieved satisfactory sensitivity and specificity in diagnosing intestinal TB. Histopathology, revealing granulomas with caseating necrosis, Langerhans giant cells, conglomerate epithelioid histiocytes, and disproportionate submucosal inflammation, is considered the gold standard for diagnosing intestinal TB. Cicatrization after intestinal ulcer healing may explain the pathological changes of the disease, while the paradoxical phenomenon of disease deterioration after ATT could also be elucidated. Intestinal TB and lymphoma are characterized by chronic granulomas. If pathological examination reveals both non caseating granulomas and submucosal lymphocyte aggregation, a diagnosis of lymphoma should be considered. Patients with intestinal lymphoma commonly present with intestinal hematochezia and perforation, whereas these manifestations are rare in intestinal TB patients.^[28]^ In our case, histopathological examination confirmed inflammatory exudates and proliferative granulomatous tissue on the surface of ileal ulcers, along with multifocal caseating necrosis accompanied by Langhans giant cells in the lymph nodes, suggesting a pathological basis for the diagnosis of intestinal TB.

The gold standard for diagnosing intestinal TB is the culturing of the *M tuberculosis* pathogen, as it exhibits very high specificity (100%); however, this method is associated with a low sensitivity value (9.3%).^[29]^ Therefore, other techniques are often employed in suspected cases. AFB staining exhibits high specificity (100%) but low sensitivity (17% to 31%), resulting in a high risk of false-negatives. PAS staining reacts with glycoproteins that are characteristic of *M tuberculosis* and thus can improve specificity and sensitivity. GMS staining, widely used for cell morphological examination, displays cell morphological characteristics such as organelles, cell layers, and free particles, but it can also reveal the presence of small intracellular bacteria. In the present case, due to suspicion of TB infection, microbial culturing, AFB staining, PAS staining, and GMS staining were all used to attempt to observe the presence of *M tuberculosis* in the lymph nodes, but all of the tests were negative. Notably, we were aware that these negative tests did not entirely rule out the possibility of TB infection, as false negatives or other factors may have affected the test results. Due to the possibility of false negative results, we comprehensively considered clinical manifestations, medical history, and other findings and thus proceeded to perform additional tests for TB.

Multiplex-PCR can be more highly sensitive than microbiological examination, and the specificity of this technique is close to 100%. In the case of *M tuberculosis,* PCR can provide a quantitative determination of the amount of bacteria present in a tissue in terms of the number of copies of the genome per milliliter of sample. High concentrations, even >500 copies/mL, can be seen in patients with TB of the chest, the intestines, the bone, the kidney, or the lymphatic system. Because it is a rapid technique, PCR-based detection of *M tuberculosis* can help clinicians quickly and accurately diagnose TB, reducing disease progression and spread. In fact, in some cases, PCR can identify the presence of *M tuberculosis* DNA even in cases where bacterial culture results are negative. However, PCR testing for *M tuberculosis* strongly influenced by sample quality and operating techniques, necessitating consideration of results from other detection methods. In our case, PCR testing for *M tuberculosis* was positive (>500 copies/mL), indicating the presence of TB infection, despite other negative test results.

The surgical approach for a patient with tuberculous intestinal perforation is complex and depends on various factors, including the patient’s overall condition, the extent of gastrointestinal involvement, and the number of perforations. Intestinal perforation is a serious and potentially fatal complication, necessitating prompt surgical intervention.^[30]^ The mortality rate associated with perforation ranges from 30% to 40%. In cases without prolonged peritoneal soilage, resection with primary anastomosis or diversion is recommended to avoid the high risk of reperforation and fistulization associated with simple closure.^[31]^ During surgery, if intestinal anastomosis is not feasible, the abdomen may be left open with the intestinal tube covered by a mesh. A Lee double cannula is then placed in the abdominal cavity for continuous irrigation and negative pressure drainage. This approach reduces the risk of intrabdominal leaks, peritonitis, or collections that may require further surgeries. It also allows for potential re-exploration in the intensive care unit, helps prevent compartment syndrome, and avoids trauma from repeated wound closure.^[32]^

In a study by Limpin et al^[33]^ surgical treatment was performed on 164 patients with gastrointestinal TB. A total of 37 patients underwent resection with an anastomosis, while 127 patients underwent diversion. In emergency surgery, ostomies were preferred for patients with severe abdominal contamination, significant intestinal dilation, and poor nutrition. Primary anastomosis is considered for patients who are well-prepared for surgery, young, with stable hemodynamics before and during surgery, good nutritional status, and controlled comorbidities. Dudaka et al^[10]^ suggested that in patients with intestinal perforation and strictures, the strictured segment should be resected. Indications for anastomosis include minimal peritoneal contamination and edema-free bowel. However, these patients may be at risk of re-leak or new perforations. Grieco et al^[34]^ recommended delayed closure of the abdomen for patients with severe abdominal contamination and unstable TB conditions, although the optimal closure method and TB suture technique remain controversial. Surgical bowel resection is suitable for multiple adjacent perforations, while primary repair is considered for single or multiple distant perforations. Small tract or wedge resection is less common but may be necessary in certain cases, with techniques varying based on the specific circumstances. Cases that recommended surgical techniques of bowel resection/repair are summarized in Table 2.^[33,35–39]^

**Table 2:** Choice of surgical approach in the patient with tubercular intestinal perforation.^[33,35–39]^

No.	Author(s) year	Patients	Resection anastomosis	Repair ileostomy
1	Pujari^[35]^ 1979	79	26	53
2	Ecgleston^[36]^ 1983	21	16	5
3	Ara^[37]^ 2005	12	5	7
4	Munghate^[38]^ 2015	48	16	32
5	Neupane^[39]^ 2022	60	7	53
6	Limpin^[33]^ 2023	164	37	127

Due to the risk of surgical site infections, which occur in more than 50% of patients who undergo surgical treatment, postoperative complications include wound dehiscence (27%), intra-abdominal collections (10%), and enterocutaneous fistula (10%).^[10]^ Conversely, laparoscopy is a rapid, safe, and effective technique for diagnosing abdominal TB, providing minimally invasive access to the peritoneal cavity. The typical laparoscopic findings of peritoneal TB comprise multiple yellowish-white tubercles spread over the peritoneum and other viscera, omental thickening with ascites, fibrous bands and adhesions, and an abdominal cocoon with matted small bowel.^[40]^ Eventually, 25% to 75% of patients with abdominal tuberculosis will require surgery, with indications including intestinal occlusion (15% to 60%), perforation (1% to 15%), abscesses and fistulas (2% to 30%), and hemorrhage (2%).^[41]^ Mini-laparoscopy is even less invasive than traditional laparoscopy, using ultra-fine instruments and can be safely performed outside the operating room while the patient is awake. Brehm et al^[23]^ reported that out of 49 consecutive patients who underwent mini-laparoscopy for suspected abdominal TB, the diagnosis was subsequently confirmed in 29 patients (59%). Therefore, tissue biopsy under diagnostic laparoscopic exploration remains the gold standard for diagnosing abdominal TB.

In our case, the patient developed perforation of the digestive tract in the context of severe anemia and malnutrition. Due to extensive abdominal adhesions that were difficult to separate laparoscopically, we converted to open surgery. Considering the patient’s poor nutrition (hypoproteinemia) and a relatively short small intestine (3.4 m), along with heavy abdominal contamination, we chose ileal repair plus ostomy. Even if intestinal perforation were to occur again for the patient, we would perform timely intestinal repair and place Lee double cannula in the abdominal cavity for continuous abdominal irrigation and negative pressure drainage. The abdominal cavity was semi-open, and the operation was completed.

This case demonstrates the importance of multidisciplinary teamwork in treating complex patients. Multiple teams were involved in the patient’s care, including TB specialists, general surgeons, hematologists, pharmacists, and intensive care teams. Older age, poor nutritional status, and unstable comorbid conditions are factors noted to contribute to both morbidity and mortality, with no major ATT-induced complications recorded.^[34]^ A long course of ATT was used for 6 months in this case. Overall, with standard treatment, high cure rates (>95%) can be achieved in patients with intestinal TB, if drug selection is appropriate, patients are compliant, and the dose and duration are adequate.^[27]^ In our case, after 15 days of ATT, the patient’s condition was stable, and he was successfully discharged. The jejunal nutrition tube was removed after 2 months. The patient was followed up for 6 months, during which his body weight increased to 55 kg. There was no liver damage observed, and the ileostomy was restored 5 months postoperatively. After 12 months of follow-up, the patient’s body weight increased to 65 kg, PCR testing was negative for TB, and anti-TB drugs were discontinued.

## 4. Conclusion

This case report describes a patient who experienced small bowel perforation due to intestinal TB. A thorough evaluation of clinical features, imaging findings, pathological characteristics, and management, is recommended for small bowel perforations due to intestinal TB. Diagnosis of this disorder remains challenging even for experienced clinicians, and the surgical treatment techniques continue to be optimized. Multiple teams were involved in the patient’s care during treatment, highlighting the importance of multidisciplinary collaboration.

## Acknowledgments

We would like to thank Prof Ding Lianan, Department of General Surgery in Affiliated Hospital of Qingdao Medical College, Qingdao University for our guidance and help to us in the operation of this study.

## Author contributions

**Data curation:** Wei Dong.

**Formal analysis:** Wei Dong.

**Funding acquisition:** Yuxu Zhong.

**Investigation:** Jianhua Ju, Jingyu Liu, Haibo Chu.

**Methodology:** Haibo Chu.

**Project administration:** Haibo Chu.

**Writing – original draft:** Jianhua Ju, Jingyu Liu.

**Writing – review & editing:** Yuxu Zhong.
